# 834. A Single-center Experience of Cardiovascular Histoplasmosis: An Emerging Complication of Histoplasmosis

**DOI:** 10.1093/ofid/ofad500.879

**Published:** 2023-11-27

**Authors:** Eloy E Ordaya, Sarah A Lawrence, Elyse L Conley, Ratnasari Padang, Mark Enzler

**Affiliations:** Mayo Clinic, Rochester, Minnesota; Mayo Clinic, Rochester, Minnesota; Mayo Clinic Rochester, Rochester, Minnesota; Mayo Clinic, Rochester, Minnesota; Mayo Clinic College of Medicine, Rochester MN, Rochester, Minnesota

## Abstract

**Background:**

Histoplasmosis is an endemic mycosis associated with a wide range of clinical presentations. Cardiovascular histoplasmosis (CVH) was rarely seen at our center before 2007 but has increased in frequency over the last 15 years. Herein, we describe the clinical characteristics of our recent CVH cases.

**Methods:**

We identified all adults diagnosed with CVH at Mayo Clinic in Rochester, MN, between May 2007 and February 2023.

**Results:**

Ten cases of CVH were identified, all of whom resided in the Midwest (Table). The majority were male, with a median age of 72 years. Five patients had prosthetic valves, two endovascular grafts, one a prosthetic valve and aortic graft, and one was a heart-kidney transplant recipient. Patients most frequently presented with prolonged night sweats, fever, and dyspnea. Cytopenias, pulmonary nodules, and splenomegaly were common laboratory and imaging findings. The diagnosis was suspected based on a positive *Histoplasma* antibody (100%) and/or antigen (80%) testing and confirmed by positive culture (blood 60%, explanted valve/graft 20%) and/or histopathology (60%). Identified clinical syndromes included: prosthetic valve endocarditis (PVE; 50%), native valve endocarditis (10%), mycotic aortic aneurysm (10%), aortic graft infection (10%), PVE and aortic graft infection (10%), and myocarditis (10%). Most patients received combined medical and surgical treatment (50%). Three patients (30%) received medical treatment only, including one patient with myocarditis. Two patients (20%) presented progressive clinical decline and were not considered antifungal treatment or surgical candidates. The overall mortality within one year of diagnosis was 50%. Patients with endocarditis and/or graft infection who received only medical treatment had higher mortality than those who received combined medical and surgical treatment (100% vs 20%, respectively).

**Table.**
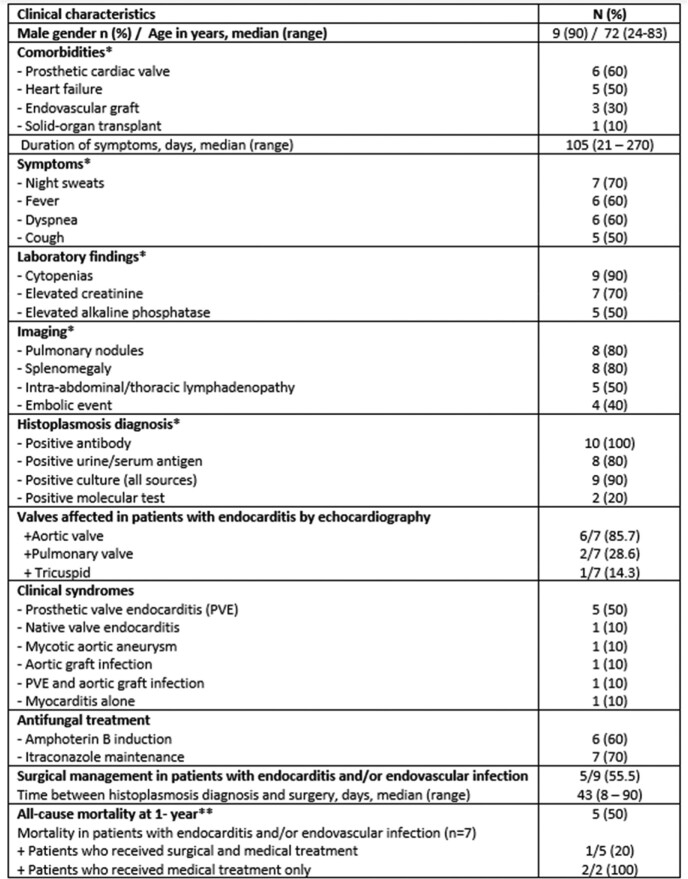
Clinical characteristics of patients presenting with cardiovascular histoplasmosis (N=10)

*Patient frequently had more than one comorbidity, symptom, laboratory or imaging finding, positive histoplasmosis testing, or affected valve. **Two patients did not receive treatment given their critical status, and were transitioned to comfort care.

**Conclusion:**

CVH is being seen more frequently at our institution. One-year mortality of CVH was high, but combined medical and surgical management conferred better outcomes than medical treatment alone. We suggest a high index of suspicion of CVH in patients residing in *Histoplasma* endemic regions with a history of prior cardiac or vascular surgery presenting with prolonged fevers and/or new cardiomyopathy.

**Disclosures:**

**All Authors**: No reported disclosures

